# The Contagion of Donation Behaviors Changes Along With the Abatement of the COVID-19 Pandemic: An Intertemporal Survey Experiment

**DOI:** 10.3389/fpsyg.2021.585128

**Published:** 2021-04-29

**Authors:** Shuaiqi Li, Xiaoli Liu, Jianbiao Li

**Affiliations:** ^1^School of Finance, Shandong University of Finance and Economics, Jinan, China; ^2^Institute for Study of Brain-like Economics/School of Economics, Shandong University, Jinan, China; ^3^Department of Economic Management, Nankai University Binhai College, Tianjin, China; ^4^Reinhard Selten Laboratory, China Academy of Corporate Governance, Nankai University, Tianjin, China

**Keywords:** COVID-19, donation, contagion of donation behavior, social anxiety, intertemporal study

## Abstract

We conducted an intertemporal online experiment to examine the contagion of others’ positive and negative donation behaviors. We collected two sets of data during and after the peak of the COVID-19 pandemic in China. The participants donated to the charitable fund, “Against COVID-19, The China Charity Federation Is on the Move.” We further investigated the mediating effect of social anxiety on the link between the contagion of donation behaviors and the changes in the COVID-19 situation. A total of 1022 participants (Mage = 22.68, 63.01% females) participated in the intertemporal online experiment and were considered in the statistical analyses. Our findings were as follows. First, the donation behaviors of others significantly changed these participants’ initial donation decisions, with increased or decreased donation amounts being associated with a positive or negative donation behavior, respectively. Others’ positive donation behavior was more likely to nudge these participants into changing their initial decisions (31.82%, Mean = 15.177, SD = 1.586) than negative donation behavior (18.28%, Mean = 12.122, SD = 1.908) during the peak of the pandemic. However, such difference disappeared after the peak because the contagion of positive donation behavior significantly decreased along with the abatement of the pandemic. Second, the participants’ social anxiety decreased along with the abatement of the pandemic, and social anxiety completely mediated the relationship between the pandemic abatement and the decrease in the contagion of positive donation behaviors. These findings advance our understanding of the motivations and influence mechanism of individuals’ donation decisions in the current pandemic situation and help make informed policy making decisions.

## Introduction

The onset of the COVID-19 pandemic at the beginning of 2020 has inspired donations from people worldwide. By the end of February 2021, the “COVID-19 Solidarity Response Fund for WHO” had succeeded in raising or securing commitments of over 242 million dollars from more than 60,000 individuals, companies, and philanthropists worldwide.^1^ Unlike donations associated with disasters, the donation channels in the context of the COVID-19 pandemic have greatly expanded as a result of the highly advanced state of the Internet, which allowed individuals to make charitable donations through convenient and extensive channels. Therefore, an examination of the mechanism that pushes individuals to donate is particularly relevant given its importance in making appropriate policy decisions.

In the current networked society, especially in the context of the COVID-19 pandemic, with high-speed information flows (Li et al., 2020), individuals can easily acquire social information about others’ behavior, which may lead them to adjust their own behavior. Previous studies have shown that individuals change their donations when they are presented with information on the donation behaviors of other donors (Shang and Croson, 2009; Hysenbelli et al., 2013; Smith et al., 2015; van Teunenbroek and Bekkers, 2020). According to van Teunenbroek et al. (2020), the influence of social information depends on three dimensions, namely, “who” is the source of social information, “what” is the content of social information, and “where” are the social information and donors. Studies focusing on “who” and “what” show that individuals tend to be influenced by information obtained from others with similar attributes (Tian and Konrath, 2019), especially when such information concerns others’ positive donation behaviors (Shang and Croson, 2009; Hysenbelli et al., 2013; Smith et al., 2015).

However, the above studies have mainly focused on positive information about other donors’ donations (van Teunenbroek et al., 2020). In the real world, the public is surrounded by information on donations. Donors not only receive positive information about what others give but also receive negative information relating to how other people damage or harm charitable donations. They may even be persuaded not to donate by their peers. However, the influence of information regarding others’ negative donation behavior on individuals’ donation decisions has been largely ignored in the literature (Dimant, 2019) and is therefore the first question that we aim to address in this study. We conducted an online survey experiment to investigate how social information affects individuals’ decisions when making actual donations to the charitable fund, “Against COVID-19, The China Charity Federation Is on the Move.”^2^ In other words, we compared the degrees of contagion of positive and negative donation behaviors in the context of the pandemic.

We are mostly interested in the “where” dimension. The pandemic gives us a chance to investigate the association between changes in social contexts and the influence of social information. Specifically, we compared the degrees of contagion of donation behaviors during and after the peak of the COVID-19 pandemic. During the peak of the pandemic, social interactions were prohibited, and the failure to maintain social distancing was generally looked down by the public. When we leave our homes, we are unsure whether we should wear our masks at all times, and when we cannot avoid sneezing, we look around in fears of receiving admonishing looks. This abnormal situation of social isolation leads to increased levels of social anxiety (Kashdan et al., 2014). However, as the COVID-19 pandemic eased, the social isolation requirements were also relaxed, which also lowered the levels of social anxiety among the public. Therefore, we assessed the degree of social anxiety experienced by individuals during and after the peak of the pandemic. We also analyzed whether the influence of the pandemic on the contagion of donation behaviors is realized through the degrees of individuals’ social anxiety.

## Theoretical Background and Hypotheses Development

### Influence of Social Information on Making Decisions Regarding Donations

Previous studies have shown that individuals’ charitable donation behaviors are often driven by three motivations. The first relates to these individuals’ internal preferences and includes pure altruism and the feeling of satisfaction that accompanies donations (Ribar and Wilhelm, 2002; Null, 2011; Konrath and Handy, 2018). The second motivation relates to reputational and self-image concerns that are linked to the observability of individuals’ own donation behavior (Reinstein and Riener, 2001). The third motivation is norm compliance, which occurs in relation to the receipt of social information and has attracted increasing attention from researchers. Previous studies show that social information, especially information regarding others’ donations, can effectively change the amount of individual donations (Shang and Croson, 2009; Hysenbelli et al., 2013; Smith et al., 2015; van Teunenbroek and Bekkers, 2020). Given the manipulability of social information, this motivation is considered an effective nudge mechanism for changing donor donations (Bartke et al., 2017; van Teunenbroek and Bekkers, 2020). Previous studies have mostly examined how social information affects charitable donation behavior in two ways, namely, through the donation rate of individual donors and through the amounts of individual donations.

In their examination of donation rate, Frey and Meier (2004) found that more students donate when they are told that more than half of other students have also donated. In another study, Agerström et al. (2016) found that the donation rate increases by 27.3% when social information is presented to individuals. However, some studies found that social information does not attract more donors. Chen et al. (2010) found that when individuals are provided social information, their donation rate does not change, but their donation amounts significantly increase.

Social information significantly affects the amounts of individual donations. For example, Chen et al. (2010) and d’Adda et al. (2017) found that below-median contributors or dictators increase their voluntary contributions or donations after being informed about the median contribution of other individuals. Specifically, Croson and Shang (2013) and Shang and Croson (2009) found that providing social information increases individuals’ donations by 12.5%. van Teunenbroek and Bekkers (2020) found that the provision of social information increases the amount of individual donations by 17%. Moreover, Agerström et al. (2016) found that individuals’ donation amounts double in the presence of social information.

Other studies have explored the mechanism through which social information influences donation behavior. The prevailing view is that social information influences donation behavior through donors’ norm compliance, which affects individuals’ voluntary investment behaviors (Fehr et al., 2002), reciprocal behavior (Gächter et al., 2013; Thöni and Gächter, 2015), altruistic behavior (Fabbri and Carbonara, 2017), and trust behaviors (Mittone and Ploner, 2011). In the context of charitable donations, Goeschl et al. (2018) found that norm perception moderates the relationship between social information and donations. In other words, available information about the decisions of others creates a social norm that may largely regulate human decisions (Bøg et al., 2012; Edwards and List, 2014; Sasaki, 2019).

### Determinants of the Effectiveness of Social Information

Previous studies have discussed those factors that affect the function of social information and found that the influence of social information depends on three dimensions, namely, “who” is the source of social information, “what” is the content of social information, and “where” are the social information and donors (van Teunenbroek et al., 2020). In the “who” dimension, previous studies show that individuals tend to be influenced by information obtained from others with similar attributes (Tian and Konrath, 2019). They called this phenomenon the “peer effect,” which is significant in the context of charitable donations (Tian and Konrath, 2019). Hysenbelli et al. (2013) found that the donation amounts of Italian students greatly increase when they are given information about the large donations of other Italians than those of Germans. Another study found that even if group membership is random and temporary, the donation behaviors of group members positively influence the donation decisions of other members (Park and Shin, 2017).

Studies on the “what” dimension find that providing information about the large donations of others increases the donation amount of individuals (Agerström et al., 2016; d’Adda et al., 2017). For example, Shang and Croson (2009) showed that people pledge to donate more money when they learn about a large donation from another donor. However, the donation amounts provided by the social information must be appropriate (Croson and Shang, 2013). Information revealing excessively high or low donation amounts reduces the effect of social information and can even reduce donation amounts (Chen et al., 2010; Hysenbelli et al., 2013; Goeschl et al., 2018). While many donation-related studies have examined the impacts of positive social information on donation behavior, evidence regarding the impacts of negative social information on donation behavior remains scarce (Dimant, 2019). This issue has been explored in relation to other psychosocial and behavioral domains, such as lying (Telli et al., 2020) and corruption (Dong et al., 2012). These studies show that negative social information can also be contagious (Dimant, 2019).

### Social Anxiety and Changes in Donations During the COVID-19 Pandemic

As for the “where” dimension, previous studies show that social media maturity, cultural characteristics, societal differences, and some other social context-related factors may influence individuals’ donation behavior (Paulin et al., 2014; Ye et al., 2015; Braun, 2017). These influences are realized by changing the preference characteristics or mental states of individuals (Ferguson et al., 2015). In this study, we focus on the mechanism of the contagion of donation behaviors during the COVID-19 pandemic, which cannot be considered independently from the prevailing social atmosphere and context during the pandemic. Social distance and self-isolation are critical in preventing the spread of COVID-19 (Forte et al., 2020a). With social interactions prohibited, attending schools and universities, going to gyms, hobby classes, and parties, visiting friends, or playing team sports have become impossible. The only people with whom people can interact are their family members, housemates, or partners. This form of social isolation can significantly change individuals’ mental states (Forte et al., 2020b) and foster or even aggravate social anxiety (Kashdan et al., 2014).

Social anxiety is defined as the fear of being in social or performative contexts that entail a potential evaluation or scrutiny by other people. A heightened level of social anxiety is accompanied by distortions in information processing, including attention, memory, and response biases (Gu et al., 2020). These biases are reflected in the increased weight of negative information, which means that individuals with high levels of social anxiety are hypervigilant regarding negative social stimuli (Harrewijn et al., 2017) and view social situations in an excessively negative fashion (Kashdan, 2007). Therefore, they face difficulties in processing social feedback and expectations appropriately (Cao et al., 2015) out of fear of receiving negative social feedback (Van der Molen et al., 2014).

As previously discussed, social norms are constructed from social information. Donors willingly comply with these norms because they want to act in a socially acceptable way (Bekkers and Wiepking, 2011). Those individuals who fail to comply anticipate negative social evaluations. Given that individuals with high social anxiety expect to receive more evaluations that are negative and are more fearful of negative evaluations compared with those individuals having low levels of social anxiety, they tend to be influenced by social information. We also investigate how the contagion of donation behaviors changes along with the abatement of the COVID-19 pandemic. With the development of the real situation, isolation policies were most stringent at the peak of the pandemic and became relaxed after the abatement of the pandemic, thereby reducing individuals’ social anxiety, which in turn lead to the low contagion of donation behaviors.

Taken together, we hypothesize that information about other individuals’ positive donation behavior can promote a donor’s donation behaviors, whereas information about other individuals’ negative donation behavior can inhibit her/his donation behaviors. Furthermore, the contagion of donation behaviors decreases along with the abatement of the COVID-19 pandemic, which, in turn, decreases social anxiety.

## Methods

### Overview of the Study

The experiment was conducted on a digital online platform in Mainland China that provides functions equivalent to Amazon Mechanical Turk. We conducted the same experiment twice on April–June 2020 and February–March 2021. The COVID-19 pandemic in China reached its peak between April and June 2020 and was significantly abated between February and March 2021. We called the first period the “Peak Period” and the second the “Easing Period.” The data we obtained from these periods allowed us to conduct an intertemporal study.

The experiment was designed as a two-stage game of give-or-take dictators adapted from Dimant (2019). The participants were matched with a charity and were given the opportunity to give or take money away from this charity. They were asked whether they would change their initial donation decisions after being provided with information regarding the donation decisions of two other donors. To elicit realistic donation decisions during the COVID-19 pandemic, the participants were informed that their decisions could determine the donation amounts to be given to the charitable fund, “Against COVID-19, The China Charity Federation Is on the Move,” initiated by the China Charity Federation. Experimental conditions were set according to the donation behavior information presented to the participants. Three experimental conditions were applied, namely, the *Baseline Condition*, the *Positive Donation Condition*, and the *Negative Donation Condition*. In the *Baseline Condition*, the participants were informed that two other participants chose to deposit their donations in their private accounts while keeping their charitable accounts unchanged. In the *Positive Donation Condition*, the participants were informed that two other participants withdrew 81 experimental currency units (ECU) and 78 ECU, respectively, from their private accounts and placed these funds in separate donation accounts. In the *Negative Donation Condition*, the participants were informed that two other participants took 80 ECU and 79 ECU, respectively, out of their donation accounts and placed these funds in separate private accounts.

### Participants

A total of 1079 participants were recruited through WeChat to take part in the online experiment. Among these participants, 724 engaged in the experiment between April and June 2020, whereas 355 engaged in the experiment between February and March 2021. Meanwhile, 58 participants were excluded from the analysis because of incomplete information or obvious errors in their responses. In sum, 94.72% of the respondents (1022 out of 1079) were considered for the statistical analyses. In the experiment conducted between April and June 2020, 138 participants engaged in the *Baseline Condition*, 283 engaged in the *Positive Donation Condition*, and 279 engaged in the *Negative Donation Condition*. By contrast, in the experiment conducted between February and March 2021, 47 participants engaged in the *Baseline Condition*, 164 engaged in the *Positive Donation Condition*, and 111 engaged in the *Negative Donation Condition*.

### Materials and Procedure

The participants were informed beforehand that the experiment involved making charitable donations to the charitable fund, “Against COVID-19, The China Charity Federation Is on the Move.” They were also asked to fill out an informed written consent provided on the platform. The research protocol was approved by the Ethics Committee of the Shandong University. The participants were then sequentially presented with the following experimental stages, which were the same across all conditions:

Stage 1: Entry of personal information. At the beginning of the experiment, the participants were requested to provide their personal information, including their gender, place of origin, number of siblings, occupation (student or non-student), highest major degree (according to discipline), study/work area, and age. To avoid endogenous influences, information regarding the participants’ political affiliations, average monthly family income, and individual average monthly expenditure were provided after the experiment.

Stage 2: Reading of experimental instructions. The participants were asked to read the experiment instructions and were informed that they were likely to be paid real money (RMB) based on their payoffs in the experiment and that their decisions would remain anonymous. They were also told that the experiment involved charitable donations made to “Against COVID-19, The China Charity Federation Is on the Move.” At the beginning of the experiment, each participant was provisionally assigned a private account with 100 ECU and a charitable account with 100 ECU. The participants were asked to choose one of the following options:

(1) Take part or all of the money from the charitable account.
(2) Transfer part or all of the money in the personal account to the charitable account.
(3) Keep the division of the money in the two accounts unchanged.

The participants were also informed that 10 of them would be randomly chosen, and only the decisions of these selected participants would be considered relevant to the payoffs and would determine their own and the charity’s payoffs. Those participants who were not selected would receive 3 RMB regardless of their decisions about the charity. If chosen randomly, these participants’ decisions would be implemented and would count toward the charity’s funds (i.e., their decisions would influence the payoffs).^3^ The experimenter would then double all of the ECUs remaining in the charitable accounts at the end of the experiment and convert them into RMB (using an exchange ratio of 10 ECU = 1 RMB). The amount would then be donated to the China Charity Federation’s charitable fund. Each participant would be provided with 3 RMB plus the money converted from the ECUs in her/his private account (using the above exchange ratio).

Stage 3: Initial decision regarding donation. The participants initially decided whether to take or give funds. If they chose one of the first two options, then they were asked to enter the corresponding amount. However, if they chose to leave the amount unchanged, then they proceeded directly to the next stage.

Stage 4: Information about others’ decisions. The participants were informed that they were randomly matched with two other participants from the database compiled for a previous experiment based on the personal information they had entered at the beginning of the experiment.^4^ They would be able to access the donation decisions of these two participants^5^ and would have an opportunity to modify their initial decisions. If they chose to modify their initial decisions, then they would enter stage 5. Otherwise, they would directly advance to stage 6.

Stage 5: Modifying the donation decision. The total amounts in the participants’ private and charitable accounts were restored to 100 ECU. The participants then made a second donation decision following the same steps described in stage 3.

Stage 6: Measurement of social anxiety. The participants were asked to complete the Social Interaction Anxiety Scale (SIAS).

After completing these six stages, the participants were asked about their political affiliations, average monthly family incomes, and individual average monthly expenditure. They were also allowed to provide their contact information by their own volition.

### Measurements

#### Dependent Variables

*Iniamount* represents the amount of money that the participant recorded in her/his initial decision. *Iniamount* = 0 if the participant chose to leave the amount unchanged, −100 ≤ *Iniamount* < 0 if the participant took some money from the charitable account, and 0 < *Iniamount* ≤ 100 if the participant placed some money into the charitable account. Meanwhile, *Secamount* represents the amount of money recorded based on the modified decision of the participants. *Secamount* = *Iniamount* if the participant did not modify her/his donation decision.

*Change* denotes whether the participant modifies her/his initial donation decision. This variable was coded “1” if the participant modified her/his donation decision and coded “0” otherwise. The change amount in the participant’s donation decision was measured by the change in the sum of money in the charitable account (*Magchange*), which was computed as the sum based on the modified decision minus the sum based on the initial decision. If the participant did not modify her/his decision, then *Magchange* takes a value of “0.” We also used the absolute value of *Magchange* as a dependent variable (*Abschange*).

The degree of individual social anxiety (*Socialanx*) was measured by the score obtained from SIAS. We applied the Chinese version of SIAS (Mattick and Clarke, 1998) to assess the degree of social anxiety. This scale includes 19 items structured on a 5-point Likert scale (from 0 = “not at all” to 4 = “extremely”). The total score in this scale ranged from 0 to 76, with a higher score indicating a more severe state of social anxiety. The Cronbach’s α was 90.4%.

#### Independent Variables

Three experimental conditions were used, namely, the *Baseline Condition*, *Positive Donation Condition*, and *Negative Donation Condition*. To facilitate the regression analysis, we created three condition dummy variables, namely, *Othersgive* and *Otherstake*. *Othersgive* was coded as “1” if the participant was in the *Positive Donation Condition* group and “0” if the participant was in the *Baseline Condition* group. *Otherstake* was coded as “1” if the participant was in the *Negative Donation Condition* group and “0” if the participant was in the *Baseline Condition* group.

*Time*, which denotes the time when the data were collected, was coded as “1” if the data were collected between February and March 2021 and “0” if the data were collected between April and June 2020.

#### Control Variables

We controlled for the personal information given by the participants during the experiment. Gender was represented by a dummy variable (*Gender*) coded as “1” if the participant was female and “0” if the participant was male. Occupation was also represented by a dummy variable (*Job*) coded as “1” if the participant was a student and “0” otherwise. We also controlled for the number of the participant’s siblings (*Brosis*), the major of her/his highest degree (*Major*; coded by discipline), her/his age at the time of the experiment (*Age*), average monthly income of her/his household (*Famincome*), and her/his average monthly personal expenditure (*Indicost*). The political affiliation of the participant, which was represented by dummy variable *Polity*, was coded as “1” if the participant was a member of the Communist Party and “0” otherwise. This variable was also controlled in the regressions.

#### Cluster Variables

We performed robust cluster regressions to test our hypotheses. Two cluster variables, namely, the place of origin (*Native*) and workplace of the participant (*Workplace*), were used in the analysis. These two variables were all coded by the provincial-level administrative region of China.

### Statistical Analyses

Descriptive analyses were conducted to describe the demographic characteristics while considering the time the data were collected (i.e., Peak Period and Easing Period).

The Kruskal–Wallis test was performed to compare the participants’ initial donation decisions (*Iniamount*) and social anxiety (*Socialanx*) among the *Baseline*, *Positive Donation*, and *Negative Donation Conditions* and between the Peak and Easing Periods. The change rates of individual decisions were also compared among different conditions and data collection times.

The Wilcoxon rank-sum test was used to test the contagion of peer behavior in the context of the COVID-19 pandemic, to compare the contagions of positive and negative donation behaviors, and to check for differences in the contagion effects of donation behaviors between the Peak and Easing Period samples.

We employed a logistic regression model and an OLS regression model to explore the contagion of others’ positive and negative donation behaviors. The regressions were all clustered by *Native* and *Workplace*. First, given that *Change*, which denotes whether a participant modified her/his initial donation decision, was a dummy variable, we performed a logistic regression to assess the effects of others’ donation behaviors on the possibility for an individual to change her/his donation behaviors. Second, we applied an OLS regression model to explore the effects of others’ different donation behaviors on the amount of change in an individual’s donation behaviors.

Third, we performed mediating effect tests based two regression models to check whether the effects of the COVID-19 pandemic severity change on the change amount and change rate of individuals’ donation behaviors were mediated by the changes in these individuals’ social anxiety.

All data were analyzed by SPSS version 19.0 and Stata 12.1.

## Results

We initially verified the comparability of different conditions and periods. Results show no significant differences in the participants’ initial donation decisions among the *Baseline*, *Positive Donation*, and *Negative Donation Conditions* for the Peak (*Iniamount*: −1.101 vs. 6.933 vs. 3.129, $\chi_{d.f\,.2}^{2}=3.910$, *p* = 0.142) and Easing Period samples (*Iniamount*: 0.894 vs. 1.946 vs. −1.500, $\chi_{d.f\,.2}^{2}=0.169$, *p* = 0.919). Moreover, no condition difference was observed for social anxiety for the Peak (*Socialanx*: 50.754 vs. 50.072 vs. 50.198,$\chi_{d.f\,.2}^{2}=0.007$, *p* = 0.997) and Easing Period samples (*Socialanx*: 43.489 vs. 42.883 vs. 43.872, $\chi_{d.f\,.2}^{2}=1.485$, *p* = 0.474). No significant differences were also observed in the participants’ initial donation decisions between these samples (*Iniamount*: 0.585 vs. 0.443, $\chi_{d.f\,.1}^{2}=0.585$, *p* = 0.443). Therefore, in a subsequent analysis, we directly compared the data for different conditions and periods.

### Demographic Characteristics

The characteristics of the participants are shown in Table 1.

**TABLE 1:** Demographic characteristics of the sample.

	Overall sample	Peak Period	Easing Period
	(*N* = 1022)	sample (*N* = 700)	sample (*N* = 322)
*Gender*	63.01% females	65.43% females	57.76%
*Age*	22.68 years (Mean)	23.56 years (Mean)	20.76 years (Mean)
*Job*	84.64% students	82.71% students	89.44% students
*Polity*	16.14% Communist	19.43% Communist	9.001% Communist
	Party	Party	Party
*Native*	30 provinces	30 provinces	30 provinces
*Workplace*	28 provinces	24 provinces	28 provinces
*Famincome*	6,715.22 RMB (Mean)	6,399.49 RMB (Mean)	7401.60 RMB (Mean)
*Indicost*	1710.13 RMB (Mean)	1760.03 RMB (Mean)	1601.64RMB (Mean)

A total of 1022 participants aged 18 to 40 years were considered in the statistical analyses (Mage = 22.68 years; 63.01% females). The places of origin of these participants covered 22 provinces, 3 municipalities, and 5 autonomous regions in China. Meanwhile, their workplaces were distributed across 20 Chinese provinces, 4 municipalities, and 4 autonomous regions. Among these participants, 84.64% were students, and 16.14% were members of the Communist Party. Their average monthly household income was 6,715.22 RMB, and their average monthly expenditure was 1710.13 RMB. Table 1 shows the demographic characteristics of the Peak and Easing Period samples.

### Analysis of the Effects of Social Information in Different Pandemic Periods

We compared *Iniamount* with *Secamount* across different experimental conditions and periods to assess the contagion of peer behavior in the context of the COVID-19 pandemic. Table 2 presents the Wilcoxon rank-sum test results of *Iniamount* and *Secamount* for the Peak (results 1–3) and Easing Period samples (results 4–6). The second and fifth results show that positive donation behavior was remarkably contagious given that the participants increased their donations after observing that their two peers transferred much money from their private accounts to their charitable accounts. Meanwhile, the third and sixth results show that negative donation behavior was also remarkably contagious. The contagion of donation behaviors was always observed in both the Peak and Easing Period samples. No significant differences were observed between the *Iniamount* and *Secamount* values in the *Baseline* condition.

**TABLE 2:** Results for the effects of social information on donation decisions.

Results	Conditions	Sample	Variables	Observations	Mean (SD)	Wilcoxon rank-sum test	
	
						*z*	*p*
1	*Baseline Condition*	Peak Period sample	*Iniamount*	138	−1.101 (2.915)	−0.057	0.955
			*Secamount*	138	−0.710 (3.304)		
2	*Positive Donation Condition*	Peak Period sample	*Iniamount*	283	6.933 (2.344)	−4.666	< 0.01
			*Secamount*	283	22.110 (2.458)		
3	*Negative Donation Condition*	Peak Period sample	*Iniamount*	279	3.129 (2.583)	3.241	0.001
			*Secamount*	279	−8.993 92.927)		
4	*Baseline Condition*	Easing Period sample	*Iniamount*	47	0.894 (4.619)	0.523	0.601
			*Secamount*	47	−2.511 (4.982)		
5	*Positive Donation Condition*	Easing Period sample	*Iniamount*	90	−1.500 (3.561)	2.428	0.015
			*Secamount*	90	10.610 (3.966)		
6	*Negative Donation Condition*	Easing Period sample	*Iniamount*	164	1.946 (5.196)	−2.061	0.039
			*Secamount*	164	−12.234 (3.977)		

We applied a logistic model to run a robust cluster regression for testing the relationships between *Change* (as the dependent variable) and the condition variables (*Others give* and *Others take* as the independent variables). We also applied an OLS model to run a robust cluster regression for testing the relationships between *Mag change* (the dependent variable) and the condition variables separately for the Peak and Easing Period samples. These regressions were all clustered by *Native* and *Workplace*. Table 3 shows the regression results including all control variables. Both the positive and negative donation behaviors of others significantly affected the participants and prompted them to modify their decisions, and the effects of negative donation behaviors on the change rate in the Easing Period sample disappeared. In general, both positive and negative donation behaviors were contagious during the Peak and Easing Periods of the COVID-19 pandemic.

**TABLE 3:** Results of regression models indicating the contagion of positive and negative donation behaviors.

Variables	Change		Magchange		Change		Magchange	
				
	Model 1 Logistic Peak Period sample	Model 2 Logistic Peak Period sample	Model 3 OLS Peak Period sample	Model 4 OLS Peak Period sample	Model 5 Logistic Easing Period sample	Model 6 Logistic Easing Period sample	Model 7 OLS Easing Period sample	Model 8 OLS Easing Period sample
*Othersgive*	1.515***		13.942***		1.015*		12.167***	
	(0.337)		(1.685)		(0.586)		(3.514)	
*Otherstake*		0.719**		−12.144***		1.160		−15.843***
		(0.347)		(2.164)		(0.822)		(5.421)
*Gender*	0.170	−0.009	0.698	2.684	−0.536	−0.139	−4.475	−2.179
	(0.237)	(0.378)	(2.577)	(2.328)	(0.393)	(0.499)	(3.121)	(4.819)
*Brosis*	−0.089	−0.005	−0.713	1.166	0.168	0.0906	1.628	−0.648
	(0.088)	(0.098)	(0.755)	(0.926)	(0.147)	(0.128)	(1.918)	(2.144)
*Job*	0.065*	−0.010	−0.421	−0.013	−0.534	−1.486	−0.213	1.010
	(0.038)	(0.053)	(0.742)	(0.645)	(0.553)	(1.096)	(5.391)	(8.220)
*Major*	−0.022	−0.041	0.608	−0.497	−0.0591	−0.0218	−0.196	0.518
	(0.044)	(0.041)	(0.650)	(0.301)	(0.0577)	(0.0711)	(0.538)	(0.834)
*Age*	0.00003	−0.003	−0.125	−0.055	0.165	0.090	1.080	−0.233
	(0.0224)	(0.026)	(0.138)	(0.291)	(0.140)	(0.155)	(1.199)	(1.239)
*Polity*	−0.100	−0.480	−0.848	0.060	0.132	0.486	0.022	−17.325
	(0.104)	(0.491)	(2.285)	(4.764)	(0.736)	(0.787)	(5.616)	(12.915)
*Famincome*	0.032*	−0.029	0.036	0.148	−0.00163	−0.051*	0.058	0.220***
	(0.018)	(0.024)	(0.298)	(0.160)	(0.0336)	(0.030)	(0.302)	(0.070)
*Indicost*	0.046	0.149	1.403	−1.454	0.00893	0.072	0.499	−0.493
	(0.124)	(0.102)	(1.468)	(1.336)	(0.122)	(0.084)	(0.945)	(0.512)
Constant	−2.551***	−2.000***	0.722	1.194	−5.057	−3.094	−21.282	5.530
	(0.459)	(0.391)	(3.056)	(5.426)	(3.222)	(3.375)	(26.082)	(29.024)
Observations	421	417	421	417	211	158	211	157
R^2^	0.070	0.025	0.074	0.048	0.063	0.046	0.056	0.085

(1) The numbers in parentheses are standard errors. (2) *p < 0.1, **p < 0.05, and ***p < 0.01. (3) The robust cluster regressions were clustered by the place of origin (Native) and workplace of the participants (Workplace).

### Comparison of the Contagion of Positive and Negative Donation Behaviors

**FIGURE 1:**
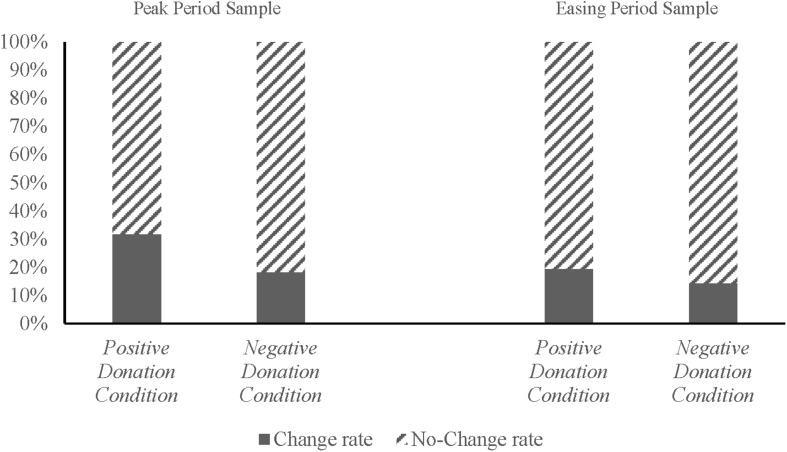
Change rates of the decisions of the participants in the positive and negative donation conditions.

Behavioral contagion depends on two dimensions, namely, the rate and amount of change of individual decisions. Our analysis of change rate revealed that for the Peak Period sample, 31.82% of the participants in the *Positive Donation Condition* group modified their initial decisions after receiving information on the positive donation behavior of their two peers, whereas only 18.28% of the participants in the *Negative Donation Condition* group modified their initial decisions. Results of the Kruskal–Wallis test show that such difference was significant ($\chi_{d.f\,.1}^{2}=13.669$, *p* < 0.01). For the Easing Period sample, 19.51 and 14.41% of the participants in the *Positive Donation Condition* and *Negative Donation Condition* groups modified their initial decisions, respectively. Results of the Kruskal–Wallis test reveal that this difference eventually disappeared ($\chi_{d.f\,.1}^{2}=1.190$, *p* = 0.275) (Figure 1), which suggests that others’ positive donation behavior, instead of their negative donation behavior, prompts the participants to modify their donation decisions during the peak of the COVID-19 pandemic. However, this phenomenon disappeared after the pandemic was eased.

**FIGURE 2:**
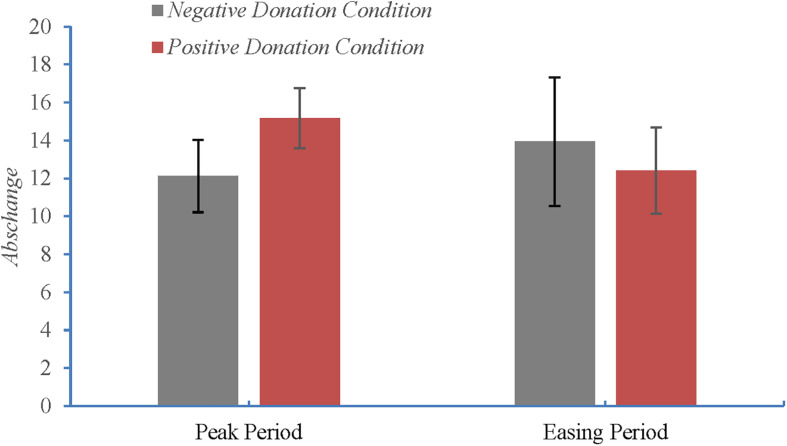
Amount of change in the participants’ donation decisions.

Table 4 reveals the change amounts in the participants’ donation decisions. Specifically, the table presents the results of the Wilcoxon rank-sum test for the *Abs change* values obtained for the Peak (result 1) and Easing Period samples (result 2) under the positive and negative donation conditions. These results, which are visualized in Figure 2, suggest that during the peak of the COVID-19 pandemic, the contagion of others’ positive donation behavior is greater than that of their negative donation behavior. However, this phenomenon disappeared when the pandemic was eased.

**TABLE 4:** Contagion of donation behaviors according to the change amounts in the participants’ donation decisions.

Result	Experimental conditions	Sample	Variables	Observation	Mean (SD)	Wilcoxon rank-sum test	
	
						Z	p
1	*Negative donation Condition* vs. *Positive Donation Condition*	Peak Period sample	*Abschange*	279	12.122 (1.908)	−3.234	0.001
			*Abschange*	283	15.177 (1.586)		
2	*Negative donation Condition* vs. *Positive Donation Condition*	Easing Period sample	*Abschange*	111	13.937 (3.396)	−0.743	0.458
			*Abschange*	164	12.415 (2.272)		

### Comparison of the Contagions of Donation Behaviors Between the Peak and Easing Period Samples

**FIGURE 3:**
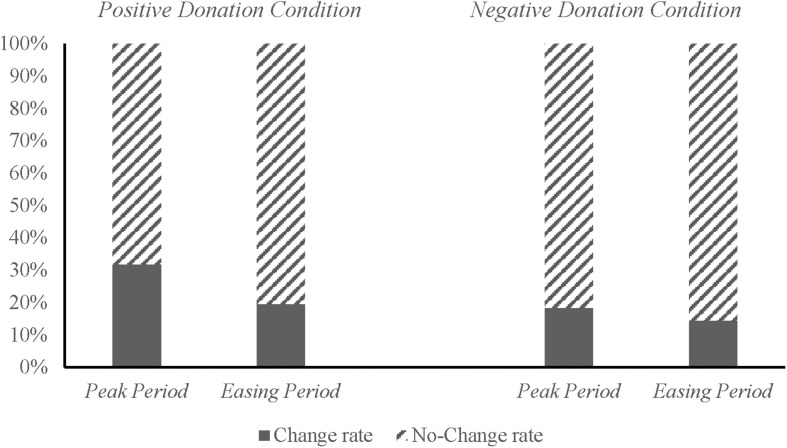
Change rates of the decisions of the participants in the Peak and Easing Periods.

Our analysis of change rate revealed that in the *Positive Donation Condition* group, 31.82% of the participants modified their initial decisions during the peak of the COVID-19 pandemic, whereas 19.51% modified their initial decisions after the easing of the pandemic ($\chi_{d.f\,.1}^{2}=7.885$, *p* < 0.01). In the *Negative Donation Condition* group, 18.28% of the participants modified their initial decisions during the peak of the COVID-19 pandemic, whereas 14.41% modified their decisions after the pandemic was eased ($\chi_{d.f\,.1}^{2}=0.832$, *p* = 0.362) (Figure 3).

Table 5 reveals the change amounts in the individuals’ donation decisions across different periods of the pandemic. The contagion of positive donation behaviors decreased along with the abatement of the pandemic (15.177 vs. 12.415, *p* = 0.0167). However, the contagion of negative donation behaviors was not affected by the abatement of the pandemic (12.122 vs. 13.937, *p* > 0.05).

**TABLE 5:** Changes in the contagion of donation behaviors along with the abatement of the COVID-19 pandemic.

Result	Time	Experimental conditions	Variables	Observation	Mean (SD)	Wilcoxon rank-sum test	
	
						z	p
1	Peak Period vs. Easing Period	*Positive Donation Condition*	*Abschange*	283	15.177 (1.586)	2.393	0.0167
			*Abschange*	164	12.415 (2.272)		
2	Peak Period vs. Easing Period	*Negative Donation Condition*	*Abschange*	279	12.122 (1.908)	0.600	0.549
			*Abschange*	111	13.937 (3.396)		

### Mediating Effects of Social Anxiety

Table 6 presents the differences in the individuals’ social anxiety across different periods of the COVID-19 pandemic. Generally, the individuals’ social anxiety decreased along with the abatement of the pandemic (50.256 vs. 43.475, *p* < 0.01).

**TABLE 6:** Changes in social anxiety along with the abatement of the COVID-19 pandemic.

Result	Variables	Conditions	Periods	Observation	Mean (SD)	Wilcoxon rank-sum test	
	
						z	p
1	*Socialanx*	Overall	Peak Period	700	50.256 (0.481)	8.006	< 0.01
			Easing Period	322	43.475 (0.768)		
2	*Socialanx*	*Baseline Condition*	Peak Period	138	50.754 (1.077)	3.263	< 0.01
			Easing Period	47	43.489 (1.632)		
3	*Socialanx*	*Positive Donation Condition*	Peak Period	283	50.198 (0.743)	4.982	< 0.01
			Easing Period	164	43.872 (1.096)		
4	*Socialanx*	*Negative Donation Condition*	Peak Period	279	50.068 (0.780)	5.276	< 0.01
			Easing Period	111	42.883 (1.375)		

We then tested whether the effects of the COVID-19 pandemic on the contagion of positive donation behaviors were realized through changes in social anxiety (see Table 7). Social anxiety completely mediated the relationship between the pandemic abatement and the decrease in the contagion of positive donation behaviors.

**TABLE 7:** Mediating effect of social anxiety in the *Positive Donation Condition*.

Variables	*Change*	*Socialanx*	*Change*	*Magchange*	*Socialanx*	*Magchange*
						
	Model 1 Logistic	Model 2 Logistic	Model 3 Logistic	Model 4 OLS	Model 5 OLS	Model 6 OLS
*Time*	−0.130***	−6.934***	−0.049	−3.834*	−6.934***	2.992
	(0.049)	(1.460)	(0.048)	(2.271)	(1.460)	(2.430)
*Socialanx*			0.012***			0.984***
			(0.002)			(0.159)
*Gender*	−0.018	1.853	−0.040	−1.412	1.853	−3.237
	(0.050)	(1.878)	(0.043)	(3.115)	(1.878)	(2.390)
*Brosis*	−0.0004	0.646	−0.008	0.444	0.646	−0.192
	(0.016)	(0.474)	(0.017)	(1.315)	(0.474)	(1.400)
*Job*	−0.074	−5.065**	−0.015	−3.716	−5.065**	1.270
	(0.085)	(1.955)	(0.080)	(4.857)	(1.955)	(4.110)
*Major*	−0.002	−0.108	−0.001	0.283	−0.108	0.389
	(0.005)	(0.152)	(0.004)	(0.392)	(0.152)	(0.351)
*Age*	−0.005	−0.371*	−0.001	−0.396**	−0.371*	−0.030
	(0.006)	(0.202)	(0.006)	(0.194)	(0.202)	(0.323)
*Polity*	0.035	0.845	0.025	1.876*	0.845	1.045
	(0.032)	(1.255)	(0.042)	(1.128)	(1.255)	(2.307)
*Famincome*	0.004	−0.003	0.004	0.173	−0.003	0.176
	(0.004)	(0.122)	(0.004)	(0.245)	(0.122)	(0.258)
*Indicost*	0.009	−0.422	0.014	2.127	−0.422	2.543**
	(0.030)	(0.657)	(0.024)	(1.704)	(0.657)	(1.273)
Constant	0.476**	62.130***	−0.248	22.12**	62.130***	−39.037***
	(0.233)	(6.673)	(0.244)	(8.705)	(6.673)	(12.852)
Observations	447	447	447	447	447	447
R^2^	0.027	0.075	0.141	0.020	0.075	0.231

(1) The numbers in parentheses are standard errors. (2) *p < 0.1, **p < 0.05, and ***p < 0.01. (3) The robust cluster regressions were clustered by the place of origin (*Native*) and workplace of the participants (*Workplace*).

## Discussion and Conclusion

Donation plays an important role in the prevention and control of COVID-19. The widespread use of the Internet and rapid development of digital technology have induced a proliferation of and an easier access to public donation channels, thereby leading to a steady rise in the importance of individual donors during the COVID-19 pandemic. An investigation of the motivations underlying individuals’ donations and their influencing mechanism is therefore critical. To shed light on this issue, we conducted an online experiment to examine how social information affects individuals’ decisions regarding donations during the pandemic. Given that people are sensitive to both positive and negative social information, we employed an adapted give-or-take donation game to study the behavioral contagion of others’ positive and negative donation behaviors. We are also interested in how the effects of social information change along with the abatement of the pandemic. We collected two sets of data in April to June 2020 (during the peak of the COVID-19 pandemic in China) and February to March 2021 (after the peak) separately.

We found that information on others’ positive donation behavior could prompt individuals to change their initial donation decisions and increase the amounts of their donations. Others’ negative donation behavior could also prompt individuals to change their decisions by decreasing the amounts of their donations. During the peak of the pandemic, the contagion of others’ positive donation behavior was greater than that of their negative donation behavior. This finding contradicts the conclusions of Dimant (2019), who reported that others’ negative donation behavior is more contagious than their positive donation behavior. These results are not surprising given that disasters often change individuals’ preferences and increase their altruism (Grimm et al., 2014) and sensitivity to positive social information (Oosterhof et al., 2009; Chen et al., 2010). However, such difference disappeared after the peak of the pandemic mainly because the cognition of positive donation behavior significantly decreased along with the abatement of the pandemic.

We attempted to explain this phenomenon by referring to the change in an individual’s mental state caused by the changes in the COVID-19 pandemic situation. Previous studies show that unlike previous pandemics, the excessively aggressive development of COVID-19 drove many countries to implement extraordinary measures to limit viral transmission, such as strict social isolation or formal mandatory quarantine (Forte et al., 2020b). These extreme rules profoundly changed the mental states of people, which is similar to what has been reported in previous studies on the SARS outbreak or other public health events (Lau et al., 2006; Zhu et al., 2008; Rubin et al., 2010). Such change in mental state was reported not only in China (Wang et al., 2020; Li et al., 2021) but also in every other country. For example, Holingue et al. (2020) found that among states with 50 or more COVID-19 cases as of March 10 in the United States, each additional day was significantly associated with an 11% increase in the odds of moving up a category of distress. Forte et al. (2020a) and Casagrande et al. (2020) found that Italians are in a state of high anxiety and demonstrate a high percentage of PTSD symptomatology. Forte et al. (2020b) and Favieri et al. (2020) found that the pandemic has similar effects on the psychopathological conditions of Italian and Chinese populations.

We focused on social anxiety as an agency of the mental states of the Chinese population given that social anxiety is sensitive to social isolation (Kashdan et al., 2014). Given the suddenness of the COVID-19 pandemic, we were unable to examine the variation tendency of social anxiety during the COVID-19 outbreak. However, we were able to record the variation tendency of social anxiety along with the abatement of the pandemic. The individuals’ social anxiety decreased along with the abatement of the pandemic, and social anxiety completely mediated the relationship between such abatement and the decrease in the contagion of positive donation behaviors. This finding offers some implications for policy makers.

Social anxiety change also had a small and insignificant effect on the cognition of negative giving behavior probably because during the peak of the COVID-19 pandemic, those donors who did not conform to negative social information only anticipated peer pressure from those individuals with negative donation behaviors. However, those donors who did not conform to positive social information anticipated peer and public pressure as well as psychological self-pressure. As the pandemic eased, those donors who did not conform to positive social information only anticipated peer pressure. The changes in pressure from positive social information were larger than that from negative social information. Therefore, the cognition of positive social information changed more than that of negative social information.

## Limitations and Future Directions

This research has several limitations. First, we only focused on information provided by peers who share similar attributes to our participants. In future studies, we plan to incorporate social network theory to explore how donors are affected by behavioral information accessed at different nodes from various sources, such as governments, non-profit organizations, and strangers. A further and expanded direction of study that can advance our understanding of the motivations and behaviors of donors entails quasi-natural experiments that explore the changes in individual preferences and the efficacy of different types of social information during the pandemic. Second, to further explain the context of the pandemic, we collected two sets of data during and after the peak of the pandemic in China. We combined these two sets of data to conduct an intertemporal investigation of how the changes in the COVID-19 pandemic situation affected the cognition of donation behaviors, which may partly interpret the context of the pandemic. However, we conducted our study after the pandemic outbreak. Therefore, we were unable to compare our recorded situations with those before the COVID-19 pandemic. Third, given that our participants were from a Chinese cultural background (Liu et al., 2019), whether our findings could be generalized to other societies with different heritage and cultural background remains an open question (Li et al., 2020). To provide solid support for the generalizability of our findings, future research should test whether these findings also apply to other cultural groups.

## Data Availability Statement

The raw data supporting the conclusions of this article will be made available by the authors, without undue reservation.

## Ethics Statement

The studies involving human participants were reviewed and approved by the Ethics Committee of the Nankai University. The patients/participants provided their written informed consent to participate in this study.

## Author Contributions

SL and JL conceived the idea of the manuscript, designed the research, and wrote the manuscript. SL and XL collected and analyzed the data and modified the manuscript. All authors have read and approved the final manuscript.

## Conflict of Interest

The authors declare that the research was conducted in the absence of any commercial or financial relationships that could be construed as a potential conflict of interest.

## Footnotes

1 For further details, see https://covid19responsefund.org/en/.

2 Detailed information can be found on the homepage of the China Charity Federation website at http://www.chinacharityfederation.org.

3 Information relating to donations can be accessed in the public information section of the China Charity Federation’s website, see http://www.chinacharityfederation.org/.

4 Previous studies show that the similarities in the attributes of the sources of social information and those of the participants determine the extent of influence of social information on these participants’ decisions (Czap and Czap, 2011; Smith et al., 2015; Tian and Konrath, 2019). We then compared the influence of positive and negative social information as well as the moderating effects of social anxiety and self-control on the influence mechanism. In the experiment, we controlled for similarities between the sources of social information and the participants.

5 In reality, these decisions were pseudo-random across the conditions applied in this experiment.

## References

[B1] Agerström J. Carlsson R. Nicklasson L. Guntell L. (2016). Using descriptive social norms to increase charitable giving: the power of local norms. *J. Econ. Psychol.* 52, 147–153. 10.1016/j.joep.2015.12.007

[B2] Bartke S. Friedl A. Gelhaar F. Reh L. (2017). Social comparison nudges—guessing the norm increases charitable giving. *Eco. Lett.* 152 73–75. 10.1016/j.econlet.2016.12.023

[B3] Bekkers R. Wiepking P. (2011). A literature review of empirical studies of philanthropy: eight mechanisms that drive charitable giving. *Nonprofit Volun. Sec. Q.* 40 924–973. 10.1177/0899764010380927

[B4] Bøg M. Harmgart H. Huck S. Jeffers A. M. (2012). Fundraising on the internet. *Kyklos* 65 18–30. 10.1111/j.1467-6435.2011.00525.x

[B5] Braun K. (2017). Decolonial perspectives on charitable spaces of “welcome culture” in germany. *Soc. Incl.* 5 38–48. 10.17645/si.v5i3.1025

[B6] Cao J. Gu R. Bi X. Zhu X. Wu H. (2015). Unexpected acceptance? patients with social anxiety disorder manifest their social expectancy in ERPs during social feedback processing. *Front. Psychol.* 6:1745. 10.3389/fpsyg.2015.01745 26635659 PMC4644791

[B7] Casagrande M. Favieri F. Tambelli R. Forte G. (2020). The enemy who sealed the world: Effects quarantine due to the COVID-19 on sleep quality, anxiety, and psychological distress in the Italian population. *Sleep Med.* 75 12–20. 10.1016/j.sleep.2020.05.011 32853913 PMC7215153

[B8] Chen Y. Harper F. M. Konstan J. Li S. X. (2010). Social comparisons and contributions to online communities: a field experiment on movielens. *Am. Eco. Rev.* 100 1358–1398. 10.1257/aer.100.4.1358

[B9] Croson R. Shang J. (2013). Limits of the effect of social information on the voluntary provision of public goods: evidence from field experiments. *Eco. Inquiry* 51 473–477. 10.1111/j.1465-7295.2012.00468.x

[B10] Czap H. J. Czap N. V. (2011). Donating-selling tradeoffs and the influence of leaders in the environmental goods game. *J. Socio Eco.* 40 743–752. 10.1016/j.socec.2011.08.004

[B11] d’Adda G. Capraro V. Tavoni M. (2017). Push, don’t nudge: behavioral spillovers and policy instruments. *Eco. Lett.* 154 92–95. 10.1016/j.econlet.2017.02.029

[B12] Dimant E. (2019). Contagion of pro-and anti-social behavior among peers and the role of social proximity. *J. Eco. Psychol.* 73 66–88. 10.1016/j.joep.2019.04.009

[B13] Dong B. Dulleck U. Torgler B. (2012). Conditional corruption. *J. Eco. Psychol.* 33 609–627. 10.1016/j.joep.2011.12.001

[B14] Edwards J. T. List J. A. (2014). Toward an understanding of why suggestions work in charitable fundraising: theory and evidence from a natural field experiment. *J. Public Eco.* 114 1–13. 10.1016/j.jpubeco.2014.02.002

[B15] Fabbri M. Carbonara E. (2017). Social influence on third-party punishment: an experiment. *J. Eco. Psychol.* 62 204–230. 10.1016/j.joep.2017.07.003

[B16] Favieri F. Forte G. Tambelli R. Casagrande M. (2020). The italians in the time of coronavirus: psychosocial aspects of unexpected COVID-19 pandemic. *SSRN Elec. J.* 8, 1–20. 10.2139/ssrn.3576804 PMC8039140 33854444

[B17] Fehr E. Fischbacher U. Gächter S. (2002). Strong reciprocity, human cooperation, and the enforcement of social norms. *Hum. Nat.* 13 1–25. 10.1007/s12110-002-1012-7 26192593

[B18] Ferguson R. Gutberg J. Schattke K. Paulin M. Jost N. (2015). Self-determination theory, social media and charitable causes: an in-depth analysis of autonomous motivation. *Eur. J. Soc. Psychol.* 45 298–307. 10.1002/ejsp.2038

[B19] Forte G. Favieri F. Tambelli R. Casagrande M. (2020a). COVID-19 pandemic in the italian population: validation of a post-traumatic stress disorder questionnaire and prevalence of PTSD symptomatology. *Int. J. Environ. Res. Public Health* 17:4151. 10.3390/ijerph17114151 32532077 PMC7312976

[B20] Forte G. Favieri F. Tambelli R. Casagrande M. (2020b). The enemy which sealed the world: effects of COVID-19 diffusion on the psychological state of the italian population. *J. Clin. Med.* 9:1802. 10.3390/jcm9061802 32531884 PMC7356935

[B21] Frey B. S. Meier S. (2004). Social comparisons and pro-social behavior: testing” conditional cooperation” in a field experiment. *Am. Eco. Rev.* 94 1717–1722.

[B22] Gächter S. Nosenzo D. Sefton M. (2013). Peer effects in pro-social behavior: social norms or social preferences? *J. Eur. Eco. Assoc.* 11 548–573. 10.1111/jeea.12015 28553193 PMC5443401

[B23] Goeschl T. Kettner S. E. Lohse J. Schwieren C. (2018). From social information to social norms: evidence from two experiments on donation behaviour. *Games* 9:91. 10.3390/g9040091

[B24] Grimm A. Hulse L. Preiss M. Schmidt S. (2014). Behavioural, emotional, and cognitive responses in european disasters: results of survivor interviews. *Disasters* 38 62–83. 10.1111/disa.12034 24325239

[B25] Gu R. Ao X. Mo L. Zhang D. (2020). Neural correlates of negative expectancy and impaired social feedback processing in social anxiety. *Soc. Cognit. Affect. Neurosci.* 15 285–291. 10.1093/scan/nsaa038 32232371 PMC7236026

[B26] Harrewijn A. Schmidt L. A. Westenberg P. M. Tang A. van der Molen M. J. (2017). Electrocortical measures of information processing biases in social anxiety disorder: a review. *Biol. Psychol.* 129 324–348. 10.1016/j.biopsycho.2017.09.013 28964790

[B27] Holingue C. Kalb L. G. Riehm K. E. Bennett D. Kapteyn A. Veldhuis C. B. (2020). Mental distress in the united states at the beginning of the COVID-19 pandemic. *Am. J. Public Health* 110 1628–1634. 10.2105/AJPH.2020.305857 32941066 PMC7542294

[B28] Hysenbelli D. Rubaltelli E. Rumiati R. (2013). Others’ opinions count, but not all of them: anchoring to ingroup versus outgroup members’ behavior in charitable giving. *Judgm. Decis. Mak.* 8, 678–690.

[B29] Kashdan T. B. (2007). Social anxiety spectrum and diminished positive experiences: theoretical synthesis and meta-analysis. *Clin. Psychol. Rev.* 27 348–365. 10.1016/j.cpr.2006.12.003 17222490

[B30] Kashdan T. B. Goodman F. R. Machell K. A. Kleiman E. M. Monfort S. S. Ciarrochi J. (2014). A contextual approach to experiential avoidance and social anxiety: Evidence from an experimental interaction and daily interactions of people with social anxiety disorder. *Emotion* 14:769. 10.1037/a0035935 24749634 PMC4191827

[B31] Konrath S. Handy F. (2018). The development and validation of the motives to donate scale. *Nonprofit Volun. Sec. Q.* 47 347–375. 10.1177/0899764017744894

[B32] Lau J. T. Yang X. Tsui H. Y. Pang E. Wing Y. K. (2006). Positive mental health-related impacts of the SARS epidemic on the general public in hong kong and their associations with other negative impacts. *J. Infect.* 53 114–124. 10.1016/j.jinf.2005.10.019 16343636 PMC7132442

[B33] Li L. Zhang Q. Wang X. Zhang J. Wang T. Gao T. L. (2020). Characterizing the propagation of situational information in social media during covid-19 epidemic: a case study on weibo. *IEEE Trans. Comput. Soc. Syst.* 7 556–562. 10.1109/TCSS.2020.2980007

[B34] Li W. Q. Li L. M. W. Jiang D. Liu S. (2021). Fate control and ingroup bias in donation for the fight with the coronavirus pandemic: the mediating role of risk perception of COVID-19. *Personal. Indiv. Differ.* 171:110456. 10.1016/j.paid.2020.110456 33071411 PMC7552985

[B35] Liu S. S. Morris M. W. Talhelm T. Yang Q. (2019). Ingroup vigilance in collectivistic cultures. *Proc. Natl. Acad. Sci.* 116 14538–14546. 10.1073/pnas.1817588116 31249140 PMC6642384

[B36] Mattick R. P. Clarke J. C. (1998). Development and validation of measures of social phobia scrutiny fear and social interaction anxiety. *Behav. Res. Therapy* 36 455–470. 10.1016/S0005-7967(97)10031-6 9670605

[B37] Mittone L. Ploner M. (2011). Peer pressure, social spillovers, and reciprocity: an experimental analysis. *Exp. Eco.* 14 203–222. 10.1007/s10683-010-9263-3

[B38] Null C. (2011). Warm glow, information, and inefficient charitable giving. *J. Public Eco.* 95 455–465. 10.1016/j.jpubeco.2010.06.018

[B39] Oosterhof L. Heuvelman A. Peters O. (2009). Donation to disaster relief campaigns: underlying social cognitive factors exposed. *Eval. Program Plan.* 32 148–157. 10.1016/j.evalprogplan.2008.10.006 19081135

[B40] Park S. Shin J. (2017). The influence of anonymous peers on prosocial behavior. *PloS One* 12:e0185521. 10.1371/journal.pone.0185521 29016612 PMC5633145

[B41] Paulin M. Ferguson R. J. Jost N. Fallu J. M. (2014). Motivating millennials to engage in charitable causes through social media. *J. Serv. Manag.* 25 334–348. 10.1108/JOSM-05-2013-0122

[B42] Reinstein D. Riener G. (2012). Reputation and influence in charitable giving: an experiment. *Theory Decis.* 72, 221–243. 10.1007/s11238-011-9245-8

[B43] Ribar D. C. Wilhelm M. O. (2002). Altruistic and joy-of-giving motivations in charitable behavior. *J. Political Eco.* 110 425–457. 10.1086/338750

[B44] Rubin G. J. Potts H. W. W. Michie S. (2010). The impact of communications about swine flu (influenza A H1N1v) on public responses to the outbreak: results from 36 national telephone surveys in the UK. *Health Technol. Assess.* 14 183–266. 10.3310/hta14340-03 20630124

[B45] Sasaki S. (2019). Majority size and conformity behavior in charitable giving: field evidence from a donation-based crowdfunding platform in Japan. *J. Eco. Psychol.* 70 36–51. 10.1016/j.joep.2018.10.011

[B46] Shang J. Croson R. (2009). A field experiment in charitable contribution: the impact of social information on the voluntary provision of public goods. *Eco. J.* 119 1422–1439. 10.1111/j.1468-0297.2009.02267.x

[B47] Smith S. Windmeijer F. Wright E. (2015). Peer effects in charitable giving: evidence from the (running) field. *Eco. J.* 125 1053–1071. 10.1111/ecoj.12114

[B48] Telli D. Espartel L. B. Araujo C. F. Basso K. (2020). Dishonesty is contagious: Investigating the domino effect of dysfunctional customer behaviors. *Revista de Administração de Empresas* 60 7–19. 10.1590/s0034-759020200103

[B49] Thöni C. Gächter S. (2015). Peer effects and social preferences in voluntary cooperation: a theoretical and experimental analysis. *J. Econ. Psychol.* 48, 72–88. 10.1016/j.joep.2015.03.001

[B50] Tian Y. Konrath S. (2019). The effects of similarity on charitable giving in donor–donor dyads: a systematic literature review. *VOLUNTAS: Int. J. Volun. Nonprofit Organ.* 2019 1–24. 10.1007/s11266-019-00165-w

[B51] Van der Molen M. J. Poppelaars E. S. Van Hartingsveldt C. T. Harrewijn A. Gunther Moor B. Westenberg P. M. (2014). Fear of negative evaluation modulates electrocortical and behavioral responses when anticipating social evaluative feedback. *Front. Hum. Neurosci.* 7:936. 10.3389/fnhum.2013.00936 24478667 PMC3896812

[B52] van Teunenbroek C. Bekkers R. (2020). Follow the crowd: social information and crowdfunding donations in a large field experiment. *J. Behav. Public Admin.* 3 1–17. 10.30636/jbpa.31.87

[B53] van Teunenbroek C. Bekkers R. Beersma B. (2020). Look to others before you leap: a systematic literature review of social information effects on donation amounts. *Nonprofit Volun. Sec. Q.* 49 53–73. 10.1177/0899764019869537

[B54] Wang C. Pan R. Wan X. Tan Y. (2020). A longitudinal study on the mental health of general population during the COVID-19 epidemic in china. *Brain Behav. Immun.* 87 40–48. 10.1016/j.bbi.2020.04.028 32298802 PMC7153528

[B55] Ye N. Teng L. Yu Y. Wang Y. (2015). “What’s in it for me?”: the effect of donation outcomes on donation behavior. *J. Business Res.* 68 480–486. 10.1016/j.jbusres.2014.09.015

[B56] Zhu X. Wu S. Miao D. Li Y. (2008). Changes in emotion of the chinese public in regard to the SARS period. *Soc. Behav. Personal. Int. J.* 36 447–454. 10.2224/sbp.2008.36.4.447

